# 2011–2021 rising prevalence of HPV infection among oropharyngeal carcinoma in France

**DOI:** 10.1186/s12885-022-10091-8

**Published:** 2022-09-20

**Authors:** Philippe Gorphe, Pierre Blanchard, Gabriel C. T. E. Garcia, Marion Classe, Caroline Even, Stéphane Temam, Ingrid Breuskin

**Affiliations:** 1 Department of Head and Neck Oncology, Gustave Roussy, University Paris Saclay, 114 rue Edouard Vaillant, 94800 Villejuif, France; 2 Department of Radiation Oncology, Gustave Roussy, University Paris Saclay, Villejuif, France; 3 Department of Radiology, Gustave Roussy, University Paris Saclay, Villejuif, France; 4 Department of Pathology, Gustave Roussy, University Paris Saclay, Villejuif, France

**Keywords:** Oropharyngeal neoplasms, Papillomavirus infections, Aging

## Abstract

**Background:**

The objective of our study was to investigate changes over the past decade in patient age and the prevalence of HPV in the population of patients with oropharyngeal carcinoma (OPC) treated at our center.

**Methods:**

We performed a retrospective cohort study of patients treated at our cancer center for OPC between 2011 and 2021. Tissue biopsies were assessed for HPV status based on p16 staining for all patients.

**Results:**

There were 1,365 treated patients. The proportion of p16-positive patients increased from 43% in 2011 to 57.3% in 2021 (*p* = 0.01). The sex ratio was 3.6 M/1F for p16-positive and 3.7 M/1F for p16-negative patients (*p* = 0.94). The mean age increased from 60.2 y in 2011 to 63.6 y in 2021. The mean ages were 61.9 y for p16-positive and 61.7 y for p16-negative patients (*p* = 0.71), but there was a broader age distribution for the p16-positive patients (*p* = 0.03). The proportion of patients older than 70 y increased from 11% in 2011 to 28.2% in 2021, and this aging was similar between p16-positive (30.7% in 2021) and p16-negative (26.3% in 2021) patients. The 2-year and 5-year OS rates were 73.7% and 56.5% for the entire cohort. p16-positive patients had 2-year and 5-year OS rates of 86.8% and 77.4%, respectively, whereas p16-negative patients had 2-year and 5-year OS rates of 63.9% and 40.5%.

**Conclusions:**

Assessment of the change over the past decade in the population of patients with OPC at our center showed that HPV-positive OPC now appear to have overtaken HPV-negative cases in France, with 57.3% in 2021, and showed significant aging, with almost thirty percent of patients now older than 70 years. Those combined changes emphasize some of the challenges to be addressed in future OPC management.

## Introduction

Head and neck squamous cell carcinomas are the sixth most frequent cancer by incidence worldwide and the fifth in France [1]. In recent decades, their incidences have slowly decreased due to reduced consumption of tobacco and alcohol in the general population [2]. Over the same period, the incidence of oropharyngeal localizations has increased in Western countries, related to the rising incidence of human papillomavirus (HPV)-driven cancers [3–7]. In France, no data are available on changes in the prevalence of HPV infection in the OPC population. However, combined changes in HPV-positive and HPV-negative OPC incidences and aging of the cancer population warrant being thoroughly examined due to their anticipated profound consequences for future standards for screening, work-up, treatment, follow-up, and prevention. For example, biological characteristics of HPV-positive OPC are used for investigative purposes in screening campaigns in at-risk populations, where circulating DNA may be a promising biomarker [8, 9]. As another example, the much lower risk of a synchronous primary head and neck cancer compared to HPV-negative OPC has been presented as a reason for omission of systematic exploration of the upper aerodigestive tract and the esophagus [10]. The favorable survival outcomes shown to occur in HPV-positive populations provide support for clinical trials to investigate treatment de-escalation aimed at decreasing long-term treatment toxicities in survivors [11–13]. The increasing number of elderly patients with HPV-positive OPC has questioned treatment optimization of locally-advanced stage disease in patients who are not candidates for cisplatin [14]. The lower risk of disease progression and recurrences and the lower risk of death from any cause in HPV-positive OPC may change follow-up guidelines [15]. Finally, the increasing prevalence of HPV infection among patients with oropharyngeal cancer may warrant intensification of national prophylactic vaccination campaigns against HPV, which was extended to include boys in France in 2021 but still struggles to reach satisfactory rates [16]. The objective of our study was, therefore, to determine the change in the OPC cancer population in the past decade.

## Materials and methods

This study was undertaken in accordance with the World Medical Association – Declaration of Helsinki – ethical principles for medical research, after having received approval from the Gustave Roussy Research Ethics Committee on the 26^th^ of November, 2020. We performed a retrospective cohort longitudinal study of patients identified from the head and neck cancer multidisciplinary committee registry database of our institution. The inclusion criteria for the current study were: patients over 18 years of age; a histologically confirmed diagnosis of squamous cell carcinoma of the oropharynx (ICD-O-3 topography codes C01.9, C02.4, C05.1, C05.2, C09.0, C09.1, C09.8, C09.9, C10.0, C10.2, C.10.3, C10.8, and C10.9); no previous history of head and neck cancer; medical management at our center from the initial stage of the cancer disease, without previous treatment in another center; available epidemiological data and follow-up data; multidisciplinary team initial discussion between the 1^st^ of January, 2011 and the 31^st^ of December, 2021. The exclusion criteria were: patients with a previous head and neck cancer localization whatever the treatment, patients who came to our center for a second opinion and were further treated elsewhere, patients referred to our center for postoperative or adjuvant treatment after an initial treatment in another center, and patients referred to our center for residual or recurrent disease after a first treatment. Of note, the population of head and neck cancer patients treated at our center comes from all geographical areas of France. In the studied cohort, 56.1% of the patients came from the Grand-Paris Ile-de-France region where is our center, which had a population of 12 million people in 2021, and 43.9% of the patients came from the rest of France.

The descriptive analysis characterized the studied population in terms of frequencies, percentages, medians, and ranges; when required the data were compared using Chi-squared tests or Fisher’s exact tests. The hypothesis underlying this study was that the population of patients treated at our center in the past ten years changed progressively during this time. Therefore, patients were stratified according to the year of the multidisciplinary team’s initial discussion, from 2011 to 2021, based on each of the epidemiological parameters analyzed. The HPV status was defined based on immunohistochemical staining for the surrogate biomarker p16-protein (CINtec p16 Histology Kit; Roche mtm laboratories AG, Heidelberg, Germany), according to the American Joint Committee on Cancer (AJCC) 2017 staging manual and the American Society of Clinical Oncology (ASCO) 2018 clinical practice guidelines [17]. Positive p16 expression was defined as strong and diffuse nuclear and cytoplasmic staining in 70% or more of the tumor cells. When evaluated, the presence of viral DNA was assessed by in situ hybridization (ISH) (Ventana HPV III Family 16, Probe B; Ventana Medical Systems, Tucson, Arizona).

Smoking history was quantified by pack-years. All head and neck CT scans of p16-negative patients were reviewed by a senior head and neck radiologist for possible diagnosis of radiological extra-nodal extension to supplement the physical examination. All cancer staging was performed using both the 7^th^ and the 8^th^ editions of the AJCC staging system. Quantitative data were compared using the Mann–Whitney–Wilcoxon test. Dispersions of quantitative data were compared using the non-parametric Ansari-Bradley test for equality of the scale parameter. Patients were stratified between the 2011–2015 and 2016–2021 time periods for survival analysis based on Kaplan–Meier curves. Overall survival was measured from the time of diagnosis to death or the last follow-up. Comparisons were performed using a log-rank test. The reported p-values were two-sided when available, and the alpha risk was five percent. The statistical analyses were performed using R software.

## Results

### Epidemiology

**Fig. 1:**
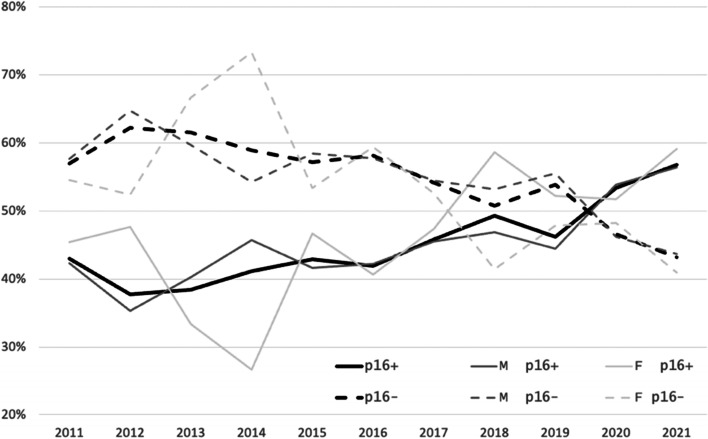
Changes in the prevalence of p16-positive and p16-negative status over time in the population of patients with oropharyngeal carcinoma (OPC)

**Fig. 2:**
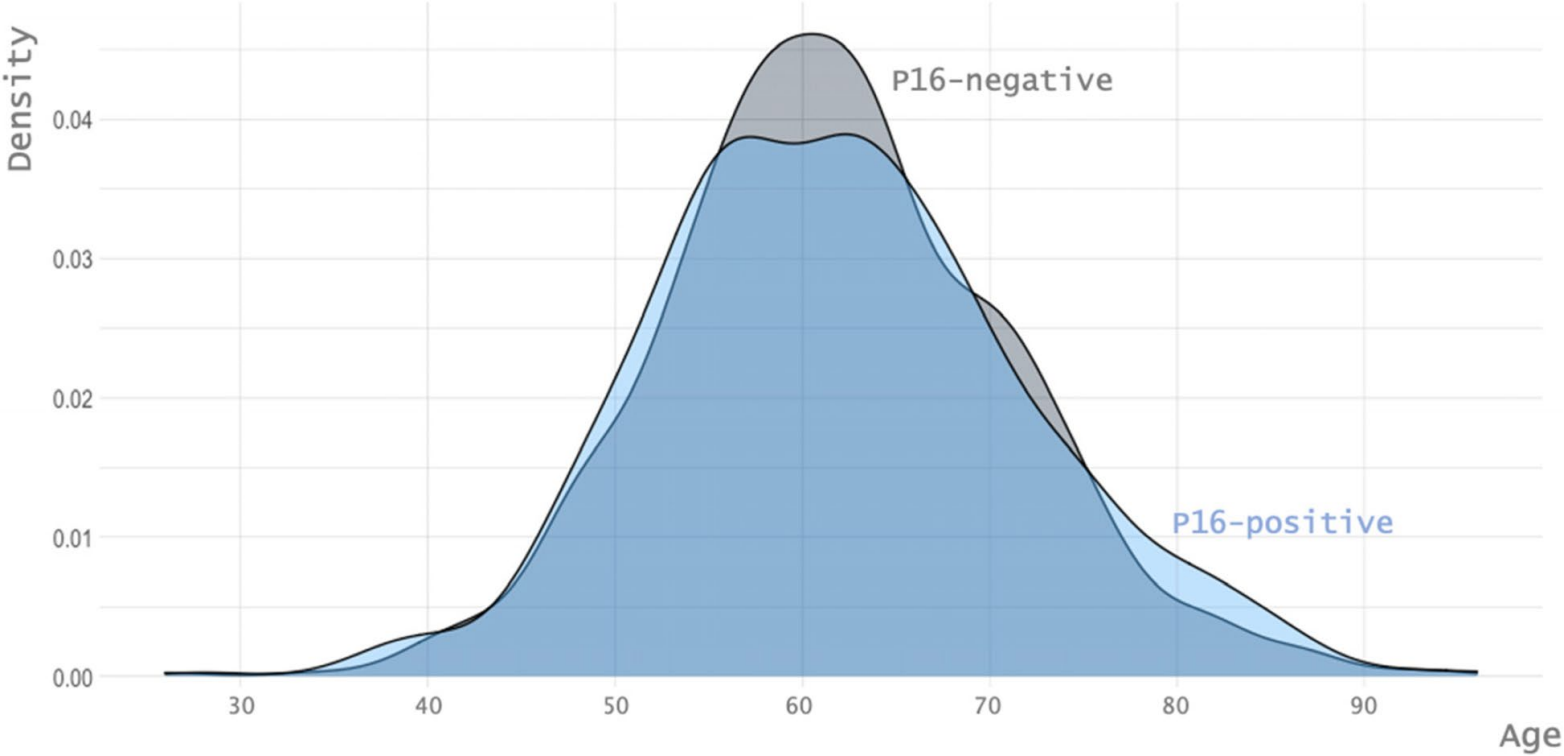
Dispersion of the age of the population of patients with oropharyngeal carcinoma, according to p16 status. The mean and the median age were similar, but the dispersions were different (Ansary-Bradley test; *p* = 0.0266), with a higher and narrower peak in p16-negative patients

**Fig. 3:**
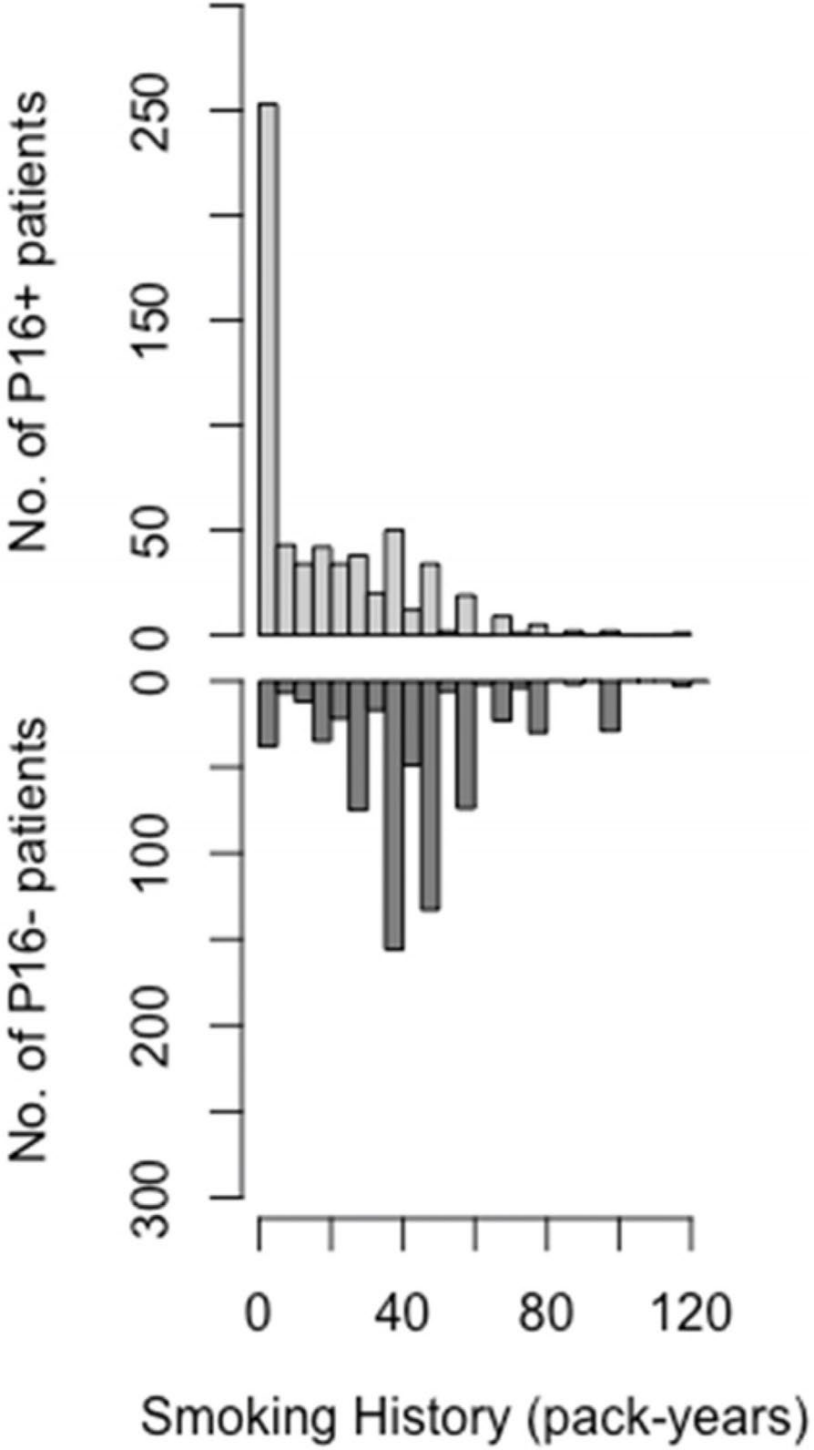
Distribution of smoking history (pack-years) in the population of patients with oropharyngeal carcinoma, according to p16 status

We identified and included 1,365 patients who fulfilled the inclusion criteria within the 2011–2021 period of time considered. Their characteristics are reported in Table 1. The HPV status was not available for 35 patients who had no tumor material for retrospective p16 status assessment; these patients were excluded from the HPV status analyses. Their characteristics are also reported in Table 1. The proportion of p16-positive patients was 45.5% among the 1,330 patients with known p16 status, and it steadily increased from 43% in 2011 to 57.3% in 2021 (*p* = 0.01) (Fig. 1). In situ hybridization was performed for 409 p16-positive patients. The rate of DNA-positive among p16-positive patients was 87%, and it did not vary over the decade studied. The proportion of women was 23.3% in the decade cohort, and it did not change significantly between 2011 and 2021 (*p* = 0.62). The sex ratio was similar between p16-positive (3.6 M/1F) and p16-negative patients (3.7 M/1F) (*p* = 0.94). The proportion of p16-positive for each sex increased similarly, from 45.4% in 2011 to 59.1% in 2021 among women, and from 42.3% in 2011 to 56.9% in 2021 among men (Fig. 1). The mean age for the overall cohort was 61.7 years of age, with no difference between p16-positive and p16-negative patients (61.9 years versus 61.7 years, respectively, *p* = 0.71). However, the age dispersion was broader among p16-positive patients (*p* = 0.03) (Fig. 2). The mean age of the population increased during the decade from 60.2 years in 2011 to 63.6 years in 2021, and this was similar for p16-positive compared to p16-negative patients as well as for men compared to women (Table 2). Notably, the proportion of patients older than 70 years of age increased from 11% in 2011 to 28.2% in 2021, and this was similar for p16-positive (30.7% in 2021) and p16-negative (26.3% in 2021) patients. The mean smoking history was 33.3 pack-years (PA) in the overall cohort population, and it was 19.4 PA in p16-positive versus 44.8 PA in p16-negative patients (*p* < 0.0001) (Fig. 3).

**Table 1:** Epidemiological and clinical characteristics of the population of patients with oropharyngeal carcinoma treated at our center between 2011 and 2021

Characteristics		No. of patients (%)			
		Total cohort	p16-positive	p16-negative	HPV-NA
Number		1365	605	725	35
Age	Mean (years)	61.7	61.9	61.7	58.3
Sex	Female	291 (21.3%)	130 (21.5%)	154 (21.2%)	7 (20%)
	Male	1074 (78.7%)	475 (78.5%)	571 (78.8%)	28 (80%)
Localization	Tonsillar fossa	582 (42.6%)	345 (57%)	222 (30.6%)	15 (42.9%)
	Tongue base	583 (42.7%)	244 (40.3*%)*	324 (44.7%)	15 (42.9%)
	Soft palate	131 (9.6*%)*	9 (1.5%)	118 (16.3%)	4 (11.4%)
	Pharyngeal wall	69 (5%)	7 (11.6%)	61 (8.4%)	1 (2.9%)
T classification (8^th^ edition)	T1	203 (14.9%)	116 (19.2%)	84 (11.6%)	3 (8.6*%)*
	T2	326 (23.9%)	175 (28.9%)	142 (19.6%)	9 (25.7%)
	T3	347 (25.4%)	148 (24.5%)	192 (26.5%)	7 (20%)
	T4	473 (34.7%)	166 (27.4%)	307 (42.3%)	16 (45.7%)
N classification (7^th^ edition)	N0	285 (20.9%)	85 (14%)	195 (26.9%)	5 (14.3%)
	N1	191 (14%)	91 (15%)	97 (13.4%)	3 (8.6%)
	N2A	88 (6.4%)	57 (9.1%)	27 (3.7%)	4 (11.4%)
	N2B	372 (27.3%)	198 (32.2%)	167 (23%)	7 (20%)
	N2C	311 (22.8%)	115 (19%)	186 (25.7%)	10 (28.6%)
	N3	118 (8.6%)	59 (9.8%)	53 (7.3%)	6 (17.1%)
N classification (8^th^ edition)	N0	-	85 (14%)	195 (26.9%)	5 (14.3%)
	N1	-	347 (57.4%)	96 (13.2%)	-
	N2	-	115 (19%)	-	-
	N2A	-	-	20 (2.8%)	-
	N2B	-	-	137 (18.9%)	-
	N2C	–	-	151 (20.8%)	-
	N3	-	58 (9.6%)	-	-
	N3A	-	-	4 (5.5%)	-
	N3B	-	-	122 (16.8%)	-
M classification	M0	1299 (95.2%)	586 (96.9%)	681 (93.9%)	32 (91.4%)
	M1	66 (4.8%)	19 (3.1%)	44 (6.1%)	3 (8.6%)
TNM stage (7^th^ edition)	Stage I	57 (41.8%)	15 (2.5%)	42 (5.8%)	0
	Stage II	77 (56.4%)	23 (3.8%)	52 (7.2%)	2 (5.7%)
	Stage III	211 (15.5%)	91 (15%)	117 (16.1%)	3 (8.6%)
	Stage IVA	767 (56.2%)	381 (63%)	371 (51.2%)	15 (42.9%)
	Stage IVB	187 (13.7%)	76 (12.6%)	99 (13.7%)	12 (34.3%)
	Stage IVC	66 (4.8%)	19 (3.1%)	44 (6.1%)	3 (8.6%)
TNM stage (8^th^ edition)	Stage I	-	118 (19.5%)	42 (5.8%)	-
	Stage II	-	157 (26%)	52 (7.2%)	-
	Stage III	-	311 (51.4%)	115 (15.9%)	-
	Stage IVA	-	-	313 (43.2%)	-
	Stage IVB	-	-	159 (21.9%)	-
	Stage IVC	-	-	44 (6.1%)	-
	Stage IV	-	19 (3.1%)		-
Performance status (WHO)	PS 0	1110 (81.3%)	541 (89.4%)	544 (75%)	25 (71.4%)
	PS 1	173 (12.7%)	47 (7.8%)	120 (16.6%)	6 (17.1%)
	PS 2–4	75 (5.5%)	15 (2.5%)	56 (7.7%)	4 (11.4%)
	NA	7 (0.5%)	2 (0.3%)	5 (0.7%)	0

**Table 2:** Change in the mean age over time in the population of patients with oropharyngeal carcinoma, according to p16 status and gender

	Overall cohort	p16 + patients	p16- patients	M	F
2011	60.2	62.3	59.0	59.7	61.8
2012	60.2	58.7	61.1	60.2	59.9
2013	58.8	57.9	59.8	57.9	61.4
2014	62.1	61.3	62.9	61.6	63.7
2015	62.4	62.8	61.9	62.2	63.0
2016	61.7	62.0	61.7	61.9	60.8
2017	61.4	61.7	61.0	61.3	61.5
2018	61.2	62.4	59.9	61.1	61.4
2019	62.3	61.8	62.7	62.0	63.3
2020	64.4	63.6	65.5	63.6	66.9
2021	63.6	63.4	63.9	63.7	63.0
Mean (SD)	61.7 (9.54)	61.9 (9.94)	61.7 (9.14)	61.5 (9.59)	62.5 (9.38)

### Disease characteristics and treatments

Between 2011 and 2021, distribution of N-classifications evolved but not T-classifications: T1-2 from 41.7 to 37.9%, T3-4 from 58.3 to 62.1%; N0 from 26.8 to 13.6%, N1 from 11.1 to 14.4%, N2A from 4.6 to 7.6%, N2B from 23.1 to 28%, N2C from 20.4 to 25.8%, and N3 from 13.9 to 10.6%. However, a PET-scan was performed in 75% of patients in 2011, and in 93.1% of patients in 2021. The rate of TNM 7^th^ edition T1–2, N0–1, M0 was 12.7% in the overall cohort, and it slowly decreased from 17.6% in 2011 to 8.3% in 2021. In 2021, out of 132 OPC patients, 88 patients with a TNM 7^th^ edition locally-advanced stage III-IV OPC were treated with curative intent with upfront radiotherapy without surgery, of whom 59 were p16-positive (67%) and 29 were p16-negative (33%). Of these, 71 patients (80.7%) were deemed to be candidates for concurrent high-dose cisplatin either weekly or three-weekly, of whom 53 were p16-positive (89.8% of the p16-positive patients) and 18 were p16-negative (62.1% of the p16-negative patients); three patients (3.4%) were given another platin drug, two of whom were p16-positive and one was p16-negative; six patients (6.8%) were given concurrent cetuximab, all of whom were p16-negative; eight patients (9.1%) were not deemed to be candidates for any concurrent drug and were treated with definitive radiotherapy alone, four were p16-positive (6.8% of the p16-positive patients) and four were p16-negative (13.8% of the p16-negative patients). Out of the 88 OPC patients treated with upfront radiotherapy in 2021, 24 were over 70 years of age, comprising 18 HPV-positive and 6 HPV-negative cases. Of the HPV-positive patients, 14 (77.8%) received high-dose cisplatin, as opposed to only 3 HPV-negative patients (50%).

### Survival

**Fig. 4:**
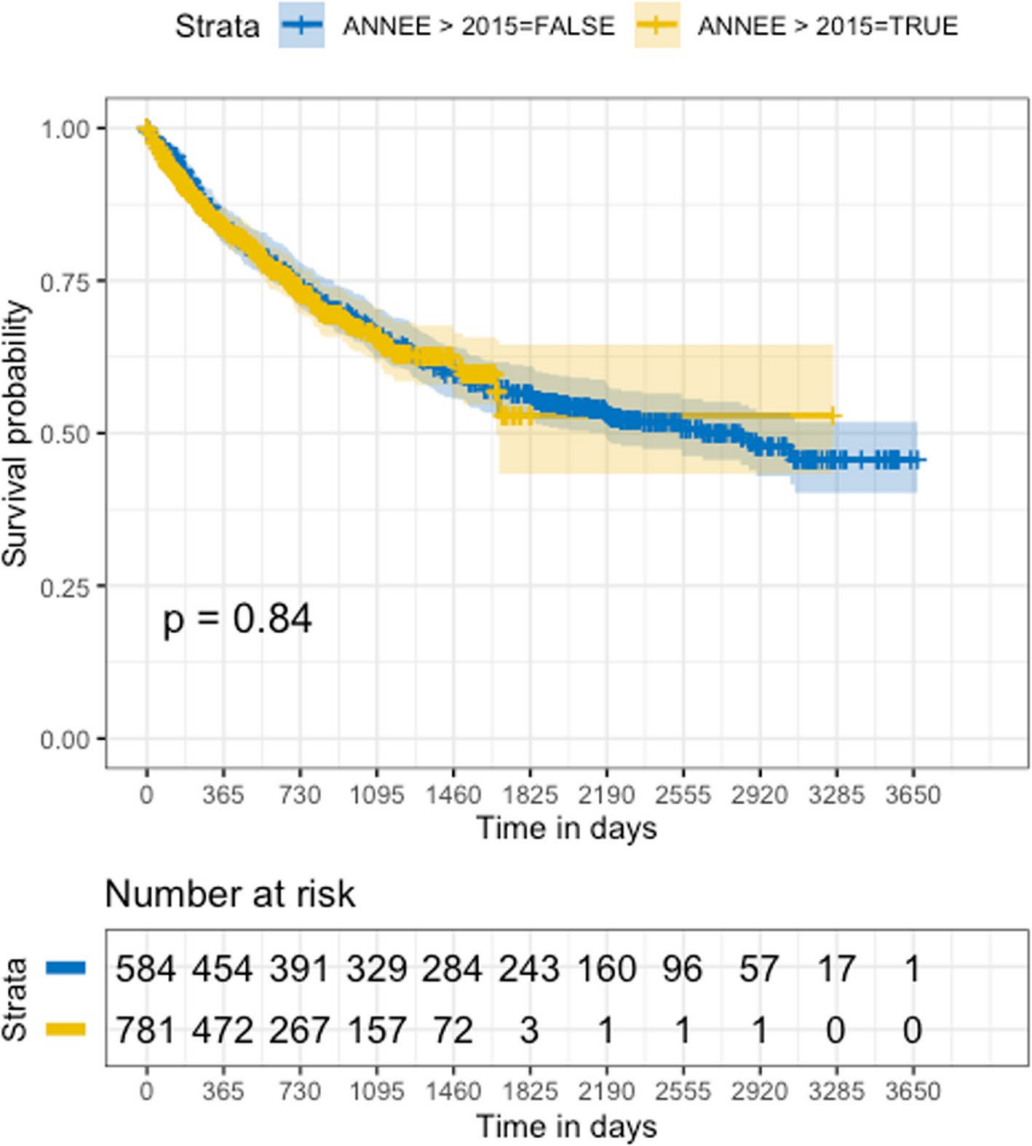
Kaplan–Meier curves of overall survival in patients treated from 2011 to 2015 and patients treated from 2016 to 2021

Median follow-up was 1224 days. The 2-year and 5-year OS rates were 73.7% and 56.5% for the entire cohort, respectively. As expected, the HPV status had a strong prognostic influence, with p16-positive patients having 2-year and 5-year OS rates of 86.8% and 77.4%, respectively, whereas p16-negative patients had 2-year and 5-year OS rates of 63.9% and 40.5%, respectively. There was no significant change in the survival of patients between the first half of the decade versus the second half of the period of time studied (Fig. 4), with 2-year OS rates of 74.2% versus 73.2%, respectively (*p* = 0.84).

## Discussion

In this study, we showed that the population of patients with oropharyngeal carcinoma (OPC) changed significantly in France in the past decade. HPV-positive OPC have now overtaken HPV-negative cases in France. The mean age of the population increased, with almost thirty percent of patients being over 70 years of age in 2021 irrespective of the HPV status. Finally, the proportion of patients potentially qualifying for single-modality treatment according to the standards in place has declined profoundly in the past decade, whereas the proportion of elderly patients qualifying for radiotherapy with concurrent high-dose cisplatin was found to be higher in HPV-positive patients. Those combined changes emphasize some of the challenges that will need to be addressed in future OPC management.

The increasing prevalence of HPV infection among patients with OPC in Europe has been shown in Sweden, Germany, the Netherlands, the United Kingdom, and Italy [3, 18–21]. At the same time, successful national health campaigns targeting heavy smoking and alcohol addictions in most developed countries over the past several decades have resulted in reduced incidences of smoking-related head and neck cancers [22]. In France, this is the first time that a study has investigated the change over time in HPV infection prevalence in OPC. The rates previously reported in France were 27.1% based on PCR DNA and mRNA positivity in 340 patients in 2009–2012, and they were between 37.7% and 43.1% based on p16-positive status with or without DNA ISH positivity in a multicenter retrospective study involving 291 patients in 2011–2012 [23, 24]. These data are in accordance with our findings of a p16-positivity rate of 43% in 2011. With the mean DNA ISH positivity of 87% that we reported in p16-positive patients, this resulted in a rate of 37.4% of DNA-positive among OPC patients in 2011. Our results confirm that the prevalence of HPV infection among OPC had continued to increase in France since these studies more than ten years ago and that HPV-positive OPC have overtaken HPV-negative cases in the past decade in France.

A large number of previous publications have reported higher rates of lymph node involvement in HPV-positive OPC than in HPV-negative cases, resulting in higher rates of locally advanced disease using the 7^th^ TNM edition [25]. The increasing prevalence of HPV infection among OPC patients in our population was concurrent with the progressive two-fold decrease of the number of patients with a 7^th^ TNM edition T1–2, N0–1 OPC in the period of time studied. These patients with early-stage disease could be candidates for a single-modality treatment either with surgery alone or with definitive radiotherapy [26]. Multi-modality treatments for patients with significant lymph node involvement according to current treatment standards in most cases comprise chemoradiotherapy when feasible with or without surgery. The increasing proportion of these patients in the OPC population argues for the development of clinical trials dedicated to reduction of treatment toxicities and improvement of patient quality of life [11–13, 27, 28].

HPV-positive OPC have been previously reported to occur in younger patients than HPV-negative OPC. Ang et al. reported a median of 53.5 and 57.0 years for HPV-positive versus HPV-negative, respectively [29]. The difference with our cohort study where we found no difference in age between HPV-negative and HPV-positive patients may be due to the selection criteria for inclusion in studies and to time-dependent age evaluation. The data of Ang et al. were based on a retrospective analysis of patients with stage III–IV 7^th^ ed. OPC included in the RTOG0129 randomized trial between 2002 and 2005 treated with high-dose cisplatin, with a subsequent selection that may have excluded older or unfit patients [29, 30]. However, time-dependent age evaluation may be an issue for comparison of published cohorts when HPV-positive and HPV-negative patients are not studied over exactly the same period of time. We showed in our study that HPV-positive as well as HPV-negative OPC patients aged linearly and with similar trends during this past decade until 2021, with nearly thirty percent of the OPC population being over seventy years. The mean age of OPC patients may continue to rise in coming years in France, and probably also in other Western countries, in line with the aging of the general population. This change in age and the higher rate of HPV-positive elderly patients who are candidates for high-dose cisplatin emphasize the importance of age as a decision variable for stage-appropriate standard of care, which should be investigated thoroughly in this specific HPV-positive population [14, 31]. It also highlights the need for the development of clinical trials dedicated to new potentializing treatments concurrent with radiotherapy in older patients who are not candidates for cisplatin [32].

Our study has some limitations that should be pointed out and kept in mind. Firstly, this was a single-center cohort study. Although our cancer center has a large recruitment area that comprises all of France, a multicenter analysis would help confirm our data. Secondly, we started our analysis in 2011 when we initiated assessment of the HPV status in OPC at our center. A retrospective analysis of OPC biopsy tissues for HPV status since 2001 would be important to confirm our findings and to more precisely document the change in HPV prevalence in France. Thirdly, we based the HPV status of the patients in our cohort on p16-protein immunohistochemical staining as this is now the international standard following the guidelines from the American Joint Committee on Cancer and the American Society of Clinical Oncology. However, DNA PCR for HPV genotyping would provide supplementary information that would help understand patterns of HPV infection in France.

## Conclusions

The population of patients with oropharyngeal carcinoma has changed significantly in France over the past decade. HPV-positive OPC have now overtaken the HPV-negative cases in France. The mean age of the population has increased, with now almost thirty percent of patients being over 70 years of age irrespective of the HPV status. Future clinical trials will have to address these new challenges, and especially the management of elderly patients.

## Data Availability

The datasets used and/or analysed during the current study are available from the corresponding author on reasonable request and with regulatory agreements.
